# Establishment of an efficient *Agrobacterium*-mediated genetic transformation protocol for *Saccharum officinarum* using Black Cheribon as a model genotype

**DOI:** 10.3389/fpls.2026.1893606

**Published:** 2026-08-25

**Authors:** Chunjia Li, Xujuan Li, Chunyan Tian, Xin Hu, Xin Lu, Gul Nawaz, Ting Yang, Xinlong Liu, Yuebin Zhang

**Affiliations:** 1 State Key Laboratory of Tropical Crops Breeding, Yunnan Academy of Agricultural Sciences, Kunming, Yunnan, China; 2 Sugarcane Research Institute, Yunnan Academy of Agricultural Sciences/Yunnan Key Laboratory of Sugarcane Genetic Improvement, Kaiyuan, Yunnan, China; 3 Key Laboratory of Southwestern Crop Gene Resources and Germplasm Innovation, Ministry of Agriculture and Rural Affairs, Kunming, Yunnan, China

**Keywords:** *Agrobacterium*, genetic transformation, *Saccharum officinarum*, sugarcane, tissue culture

## Abstract

Efficient *Agrobacterium*-mediated transformation (AMT) is vital for the biotechnological improvement of sugarcane (*Saccharum* spp.). *Saccharum officinarum* is the main ancestor of all modern cultivars, yet little research has been conducted on its AMT system. In this work, an efficient AMT protocol for *S. officinarum* was developed, with Black Cheribon as the model genotype owing to its superior tissue culture performance and regeneration capacity. The optimized agro-infection protocol comprised the following main parameters: concentration of acetosyringone (AS) in *Agrobacterium* culture, concentration of AS for infection, *Agrobacterium* concentration at OD600 = 0.4, infection time of 30 minutes, vacuum infiltration time of 10 minutes and co-cultivation time of 3 days. To further improve transformation efficiency, 0.5 mg/L thidiazuron and 200 mg/L citric acid were added to the regeneration medium, which enhanced the regeneration of shoots. A modified stage-dependent selection strategy (FlexII) was established by using glufosinate-ammonium at concentrations of 2.0, 1.0, and 0.75 mg/L in the callus proliferation, shoot regeneration, and rooting stages, respectively. This strategy was more successful than the minimum inhibitory concentration-based strategy in *S. officinarum* transformation. The optimized protocol further boosted the transformation efficiency of Black Cheribon from 1.12% to 7.17%. The resulting transgenic lines were confirmed by PCR amplification of T-DNA regions and immunochromatographic detection of *Bar* protein expression in primary transformants, respectively. These results provide a sound technical foundation for the functional genomics and biotechnological optimization of *S. officinarum* germplasm, and may serve as a reference for future transformation studies in other sugarcane germplasm.

## 1 Introduction

Sugarcane (*Saccharum* spp.) is a major crop, producing more than 80% sucrose and some 40% of the world’s bioethanol. It is cultivated in over 100 tropical and subtropical countries on an estimated 26 million hectares and is an important contributor to the livelihoods of smallholder farmers and to rural development (FAO, 2023). In this context, sustainable sugarcane cultivation plays a crucial role in addressing issues such as food security, renewable energy needs, and economic sustainability among farming communities. Breeding techniques have played a role in the sustainable production of sugarcane and have been used to continually improve sugarcane varieties (Chen et al., 2011). In recent years, climate change and consumer preferences have introduced new challenges, leading to an increased demand for elite varieties with several desirable characteristics, including yield, sucrose content, and resistance to various stresses.

These goals are difficult to achieve using cross-breeding because of the physiology and genetic complexity of the crop. Sugarcane is an auto-allopolyploid crop with a large and complex genome among the major crops (Healey et al., 2024; Zhang et al., 2025). In traditional breeding, the complexity of gene networks and high genomic redundancy (Liu et al., 2023) typically hinder the selection of desirable traits, leading to linkage drag from undesirable alleles (Yang et al., 2019). The success of crosses is further hampered by long growing cycles, asynchronous flowering among the parental lines, and problems in hybridization (Vengavasi et al., 2022; Gao and Wang, 2025). Conventional breeding is a slow process due to the limitations of genetic and physiological factors, with a breeding cycle lasting for more than 10 years (Luo et al., 2023).

However, new gene editing technologies like CRISPR/Cas9 offer tremendous potential for the development of more accurate and efficient crop improvement (Gao, 2021; Sun et al., 2025). But efficient and powerful plant genetic transformation systems are crucial for the application of the CRISPR/Cas9 system in plants. Although genotype independence is an advantage of biolistic transformation in most crops, including sugarcane (Ramasamy et al., 2018; Budeguer et al., 2021), *Agrobacterium*-mediated transformation (AMT) also has some advantages in addition to its high cost-effectiveness (Kim et al., 2016; Zhang et al., 2022). To allow for more precise integration events, AMT uses defined T-DNA borders. This mechanism usually leads to more straightforward expression patterns, a smaller number of integrated copies, and a much lower risk of disruption of the host genome and chromosomal rearrangements, in comparison to the random integration that occurs with traditional biolistics (Gelvin, 2017; Hwang et al., 2017). So, AMT has emerged as a low-cost method for the efficient production of high-quality, regulatory-friendly transgenic events in sugarcane genetic engineering (Jackson et al., 2013).

Since the establishment of a functional AMT protocol for sugarcane (Arencibia et al., 1998) many transgenic lines of the crop have been generated with interesting traits, such as resistance to the sugarcane shoot borer (*Chilo infuscatellus*) (Arvinth et al., 2010) and sugarcane streak mosaic virus (Wang et al., 2022), enhanced drought tolerance (Narayan et al., 2023), and improved agronomic characteristics such as sucrose content and biomass yield (Anur et al., 2020). A landmark study by Jung and Altpeter (2016) showed the effectiveness of AMT in the context of genome editing by comparing AMT and biolistics with the TALEN (Transcription Activator-Like Effector Nuclease) system targeting the *COMT* gene. This study, reporting higher efficiency of editing by means of AMT, marked the first application of genome editing technology in sugarcane. In recent years, AMT has proven to have a high level of effectiveness for precise genome modification using the CRISPR-Cas9 gene editing tool, such as *MTL* to increase the efficiency of haploid induction (Guo et al., 2024) and *LIM* to reduce lignin content (Laksana et al., 2024). AMT has therefore provided a simple and versatile method of transgenic and precision gene editing approaches to sugarcane breeding. As compared with other crops, however, the employment in sugarcane has been limited primarily to a small number of model cultivars that have a very high regeneration competence (Altpeter et al., 2016; Brant et al., 2025). Only limited research is available for other important sugarcane germplasm resources.

All commercial sugarcane cultivars sugarcane today are predominantly interspecific hybrids of *Saccharum officinarum* and *Saccharum spontaneum*. In these highly polyploid genomes, the proportion of the *S. officinarum* genetic contribution to the chromosomal complement is approximately 80% (Piperidis and D’Hont, 2020; Pompidor et al., 2021). Among ancestral *S. officinarum* accessions, Black Cheribon is acknowledged as a seminal accession of *S. officinarum* (Chen et al., 2011; Thirugnanasambandam et al., 2023) and is widely considered to be the founder parent of the *S. officinarum* subgenome that introgressed into global cultivars. The chromosomal structure of its auto-octaploid genome (2n = 8x = 80) is quite simple compared to the more aneuploid and variable combination found in modern hybrids (2n = 100–130) (D’Hont et al., 1998; Piperidis and D’Hont, 2020; Pompidor et al., 2021). The phenotypical characteristics of Black Cheribon are typical of the *S. officinarum* ideotype, which are moderate yield, acceptable sucrose content, high juice purity and low fiber content. It also displays high levels of agronomic sensitivity, such as low tillering, high susceptibility to major diseases (e.g., mosaic, smut) and low abiotic stress tolerance (Chen et al., 2011).

Black Cheribon is a highly suitable model genotype for functional genomics, due to its genomic simplicity. It has lower genomic complexity, which decreases the chances of interactions during characterization of a transgenic insertion or a gene-edited allele. Furthermore, it has been reported previously that Black Cheribon has good embryogenic callus induction capabilities, a high capacity for regeneration, and is more responsive to tissue culture than other *S. officinarum* accessions, including Badila (Van der Vyver, 2010) and NG77-69 (Pillay, 2013), making it an ideal choice for genetic transformation and genome editing applications.

Meanwhile, its biological vulnerability provides for stringent testing of genetic alternatives to increase resistance to biotic and abiotic stressors. Also, considering its widespread introgression into commercial cultivars, knowledge gained by using Black Cheribon as a recipient genotype is more applicable to current cultivars than from the other ancestral accessions. Despite these considerations, a robust and efficient AMT protocol for Black Cheribon or *S. officinarum* germplasm more broadly is still unavailable. Despite its strategic significance, no highly efficient and optimized AMT platform has yet been established. This gap is a serious constraint for the molecular dissection and rapid biotechnological advancements in sugarcane.

To fill this gap, this research established an efficient AMT protocol for Black Cheribon. The tissue culture response of this genotype was studied, and the effects of agro-infection, regeneration medium, and selection procedures on the efficiency of AMT were investigated. As protocol development was the primary objective, these key parameters were optimized in a stepwise manner to determine practical operating conditions for each transformation stage, ultimately yielding a robust and reproducible AMT system. The effectiveness of this system has been proven through targeted mutagenesis of *TEOSINTE BRANCHED1* using CRISPR/Cas9 technology (Huang et al., 2026). Our protocol provides an efficient tool for functional genomics of Black Cheribon, and can be employed as a reference to facilitate the precision genome engineering of other sugarcane germplasm.

## 2 Materials and methods

### 2.1 Plant growth

Black Cheribon plants were acquired from the National Germplasm Repository of Sugarcane in Kaiyuan (NGRS-KY), China. The sugarcane clones were grown under recommended agronomic management practices. Plant protection measures were not applied, as no pests or diseases were observed during the course of the experiment.

### 2.2 Medium

The composition of the media used in this study is detailed in **Table 1**. MS medium was used as the basal medium. Before sterilization via autoclaving (121 °C for 20 min), the media were adjusted to the appropriate pH levels and then poured into sterile 90-mm Petri dishes (NEST Biotechnology Co., Ltd., Wuxi, China).

**Table 1:** Composition of media for tissue culture and genetic transformation of Black Cheribon.

Medium	Components
CIM	MS + 2,4-D 3.0 mg/L + CAH 0.5 g/L + Sucrose 30 g/L + Agar 8.0 g/L, pH 5.8-5.9
RM	MS + 6-BA 2.0 mg/L + Sucrose 30 g/L + Agar 8.0 g/L, pH 5.8-5.9
RTM	1/2MS + NAA 2.0 mg/L + activated carbon 750 mg/L + Sucrose 30 g/L + Agar 8.0 g/L, pH 5.8-5.9
INCM	1/2MS + 2,4-D 2.0 mg/L + AS 100 μM* + Sucrose 10 g/L + Glucose 20 g/L, pH 5.2
CIMT	CIM + Tim 200 mg/L
CIMTP	CIM + Tim 200 mg/L + PPT 2.0 mg/L*
RMTP	RM + Tim 200 mg/L + PPT 2.0 mg/L*
RTMTP	RTM + Tim 200 mg/L + PPT 2.0 mg/L*

*Initial concentrations of AS and PPT, otherwise specified in the optimization.

Heat-stable supplements, including 2,4-dichlorophenoxyacetic acid (2,4-D), 6-benzylaminopurine (6-BA), naphthaleneacetic acid (NAA), casein hydrolysate (CAH), activated carbon, and citric acid, were added before autoclaving. Heat-labile components such as acetosyringone (AS), thidiazuron (TDZ), Timentin (Tim), and phosphinothricin (PPT) were filter-sterilized using 0.22 μm syringe filters (Jet Biofil, Guangzhou, China) and added aseptically to the autoclaved medium after cooling to 55 °C. All chemical reagents and medium components were purchased from Coolaber Science & Technology (Beijing, China).

### 2.3 Plant binary expression vector

**Figure 1:**
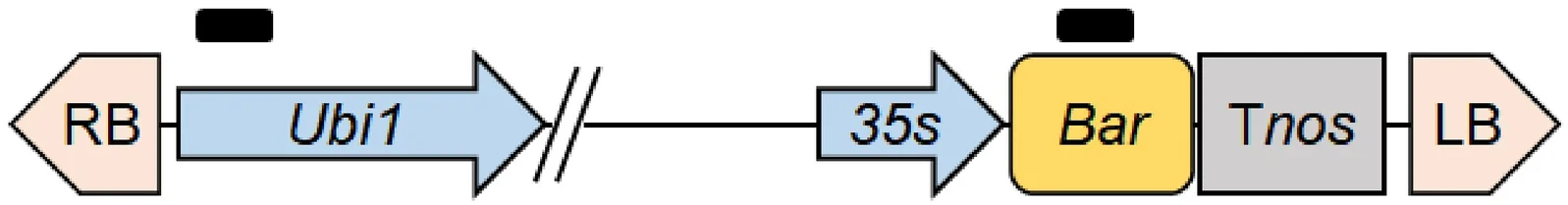
Schematic representation of plant binary expression vector pVK114. LB/RB, left/right T-DNA border; *Ubi1*, maize *ubiquitin-1* gene promoter; *35s*, cauliflower mosaic virus 35S promoter; T*nos*, nopaline synthase gene terminator; Black bars indicate probes in *the Ubi1* promoter and *Bar* gene for PCR detection of transgenes.

Plant binary expression vector pVK114 was used in this study. It harbored the gene of interest under the control of the maize (*Zea mays) ubiquitin-1* gene promoter *Ubi1*, and the herbicide-resistant marker gene *Bar* under the control of the cauliflower mosaic virus (CaMV) 35S promoter (**Figure 1**).

### 2.4 Embryonic callus induction and regeneration

Apical portions were collected from healthy stalks of field-grown plants aged 8 to 10 months. The mature leaves were removed to obtain the immature leaf whorls. These leaf whorls were surface-sterilized with 75% (v/v) ethanol and then transferred to a laminar airflow workbench. Segments approximately 4 cm in length were excised from these whorls, at a position about 1 cm above the apical meristem. Leaf discs of ~1 mm in thickness were transversely sectioned from the segments and used as explants.

For embryogenic callus induction, the leaf disc explants were cultured on callus induction medium (CIM, **Table 1**) supplemented with varying concentrations of 2,4-D (1.0, 2.0, 3.0, and 4.0 mg/L). The cultures were incubated in darkness at 28 °C and subcultured onto fresh medium every three weeks. We identified embryogenic callus using its morphological characteristics, following (Taylor et al., 1992), and tracked the induction frequency every three weeks.

For shoot regeneration, the embryogenic calli were divided into pieces ~3 mm in size and transferred to regeneration medium (RM, **Table 1**) containing different concentrations of 6-BA (0, 0.5, 1.0, and 2.0 mg/L). These cultures were incubated in a growth chamber (Bluepard Instruments Co., Ltd., Shanghai, China) at 28 °C with a 16-h light/8-h dark photoperiod and a light intensity of 80 μmol m^-2^ s^-1^. Calli with green spots or shoots were considered regenerated, and the regeneration frequency was recorded weekly. Regenerated shoots were placed in the rooting medium (RTM, **Table 1**) for rooting under the same environmental conditions used during regeneration.

### 2.5 Selection of the minimum inhibitory PPT concentrations

The PPT minimum inhibitory concentrations (MICs) were sequentially tested at three stages of development: callus proliferation, shoot differentiation and root formation. At the callus proliferation stage, embryogenic calli were transferred to CIMTP (**Table 1**) with PPT at a gradient of concentrations (0, 2, 4, 6, 8, and 10 mg/L). To monitor growth, the calli in each Petri dish were weighed to obtain an initial fresh mass at the beginning of the incubation period and again at weekly intervals. Embryogenic calli were cultured on RMTP supplemented with 0, 0.5, 1.0, 1.5, 2.0 and 2.5 mg/L of PPT at the stage of shoot differentiation. The regeneration frequency was recorded weekly. To promote root growth in the regenerated shoots, the following concentrations of PPT were used: 0, 0.25, 0.5, 0.75, 1.0 and 1.25 mg/L in the RTMTP at the root formation stage. The rooting frequency was then assessed weekly.

### 2.6 Summary of initial transformation protocol

*A. tumefaciens* strain EHA105 harboring the engineered plant binary expression vector (**Figure 1**) was spread over an LB (Luria–Bertani) agar plate supplemented with 50 mg/L kanamycin and incubated at 28 °C in the dark for 24 h. The bacterial culture was then scraped off and completely resuspended in INCM liquid (**Table 1**). The bacterial density was adjusted to an OD_600_ of 0.4, following which a 1-hour incubation was conducted at 28 °C with shaking at 100 rpm in the dark.

The calli were blotted on sterile filter paper to remove residual medium and air-dried for ~15 min under laminar airflow. The calli were then agro-infected for 20 minutes with the prepared EHA105 suspension. The infection was carried out in a dark environment with a 5-min static incubation followed by a 5-min vacuum treatment at -35 kPa and an additional 10-min static incubation. Following infection, the calli were retrieved and blotted dry again on sterile filter paper under laminar airflow for ~30 min. The air-dried calli were transferred to Petri dishes containing filter paper moistened with INCM liquid and incubated in the dark at 22 °C for 3 days of co-cultivation. After co-cultivation, the calli were washed three times with sterile distilled water and then with a 300 mg/L Timentin solution to eliminate residual *Agrobacterium*. The calli were then air-dried, transferred to CIMT medium, and incubated in darkness at 28 °C for 7 d for recovery.

For resistance screening, the calli were cultured on CIMTP for six weeks. Surviving resistant calli, with their necrotic parts removed, were transferred to RMTP for an additional six weeks to induce shoot regeneration. From each regenerating callus, a maximum of three most vigorous resistant shoots were isolated and transferred to RTMTP to generate complete plantlets with well-developed root systems. As the initial selection method, a constant PPT concentration of 2.0 mg/L was maintained in all selective media following Wang et al., 2017 (**Table 1**). Culture conditions for each of these selection stages were maintained in accordance with the standard tissue culture protocol.

Resistant plantlets were transplanted into peat soil (Pindstrup, Denmark) mixed with 20% (v/v) perlite once they reached ~4 cm in height and had developed more than three roots, each about 2 cm long. The plants were grown in a greenhouse at 28–32 °C for three weeks until they reached ~8 cm in height. To assess herbicide resistance, a diluted solution of 160 mg/L Basta (glufosinate ammonium, BASF, Germany) was applied by foliar spray. Surviving plants were sampled for molecular detection two weeks post-spraying. Confirmed transgenic sugarcane lines were transplanted to the field for further growth.

### 2.7 Optimization of the transformation protocol

#### 2.7.1 *Agrobacterium* infection

To identify conditions that maximized transformation efficiency, a one-factor-at-a-time experimental design was employed, testing six key factors. Each factor was evaluated across four levels: AS concentration in the LB plate (0, 50, 100, and 150 µM); *Agrobacterium* density (OD_600_ = 0.3, 0.4, 0.5, and 0.6); incubation duration (10, 20, 30, and 40 min); vacuumization duration (2.5, 5.0, 7.5, and 10 min); AS concentration in INCM (50, 100, 150, and 200 µM); and co-cultivation duration (2, 3, 4, and 5 d). For each factor tested, the baseline level was set according to the initial protocol described previously, while the other factors were maintained at their respective baseline levels.

#### 2.7.2 Shoot regeneration

The effects of TDZ and citric acid on shoot regeneration and transformation efficiency were investigated using a full factorial experimental design. This design consisted of three treatment combinations of TDZ concentrations (0.5, 1.0, and 1.5 mg/L) and three citric acid concentrations (100, 200, and 300 mg/L). These combinations were added to RMTP as shown in **Table 1** (hereafter called modified RMTP, or mRMTP, **Table 2**). The concentration ranges were selected based on preliminary dose-response experiments. Lower TDZ concentrations (≤0.25 mg/L) were insufficient to stimulate maximal shoot regeneration, while higher concentrations (≥1.5 mg/L) promoted excessive callus proliferation at the expense of shoot differentiation. Similarly, citric acid at concentrations of 100 mg/L was insufficient to prevent phenolic browning, and higher concentrations (300 mg/L) showed no additional benefit. Calli grown with an optimized infection protocol were transferred to these mRMTP media to determine their shoot regeneration and transformation efficiency.

**Table 2:** Effect of combinations of TDZ and citric acid at varying concentrations on regeneration performance and transformation efficiency of agro-infected calli.

Medium	TDZ (mg/L)	Citric acid (mg/L)	Regeneration frequency (%)	Regeneration index	Transformation efficiency (%)
RMTP	0	0	11.55 ± 1.75 cd	4.53 ± 0.68 d	3.38 ± 0.85 b
mRMTP1	0.5	100	13.03 ± 1.69 bc	4.40 ± 0.58 d	3.68 ± 0.61 ab
mRMTP2	0.5	200	15.94 ± 1.65 a	6.55 ± 0.47 bc	4.73 ± 0.73 a
mRMTP3	0.5	300	14.58 ± 1.21 ab	4.65 ± 0.54 d	3.86 ± 0.43 ab
mRMTP4	1	100	13.44 ± 0.82 bc	6.91 ± 0.75 b	3.30 ± 0.62 b
mRMTP5	1	200	12.15 ± 1.04 bcd	5.54 ± 0.36 cd	3.48 ± 0.14 b
mRMTP6	1	300	13.28 ± 1.26 bc	5.48 ± 0.37 cd	3.96 ± 0.52 ab
mRMTP7	1.5	100	9.89 ± 0.72 d	6.69 ± 0.83 bc	1.73 ± 0.84 c
mRMTP8	1.5	200	11.09 ± 1.40 cd	8.58 ± 1.07 a	2.04 ± 0.48 c
mRMTP9	1.5	300	11.88 ± 1.63 cd	9.10 ± 0.79 a	3.39 ± 0.83 b

Data are shown as means ± SD (n = 3). Different lowercase letters indicate significant differences at *P* < 0.05 following Duncan’s method.

#### 2.7.3 Selection method

In addition to the original selection method, three other screening procedures were developed after determining PPT sensitivity, which were Strict, FlexI, and FlexII (**Table 3**). The Strict protocol was followed without any deviation from the MIC for each step, while FlexI and FlexII were done with some flexibility relative to the MIC. These four screening methods were compared to assess their efficiency in producing transgenic events.

**Table 3:** Differences in transformation efficiency among selection methods.

Selection method	PPT (mg/L)			Transformation efficiency (%)
	Callus selection	Regeneration selection	Rooting selection	
Initial	2.0	2.0	2.0	4.51 ± 0.84 b
Strict	6.0	2.0	0.75	0.60 ± 0.52 c
FlexI	2.0	2.0	0.75	5.51 ± 0.70 b
FlexII	2.0	1.0	0.75	7.17 ± 0.74 a

Data are shown as means ± SD (n = 3). Different lowercase letters indicate significant differences at *P* < 0.05 following Duncan’s method.

### 2.8 Molecular analyses

#### 2.8.1 PCR analysis

Young leaves of putative transgenic plants were used to isolate genomic DNA using the EasyPure Plant Genomic DNA Kit (TransGen Biotech, Beijing, China). Specific DNA regions flanking the *ZmUbi1* promoter (432 bp) and the *Bar* gene (489 bp) were amplified using AceTaq Master Mix (Vazyme Biotech, Nanjing, China) with 10 ng of genomic DNA, to confirm integration of the transgenes. The amplifications were performed with gene-specific primer pairs Ubi1-F (5’-CCATCCATCATCTTCAATTCG-3’)/R (5’-CGGTAGTTCTACTTCTGTTCA-3’) and Bar-F (5’-TGCACCATCGTCAACCACTACAT-3’)/R (5’-TCAAATCTCGGTGACGGGCAG-3’), respectively. The plasmid vector, wild-type plant genomic DNA, and ddH_2_O were used as positive, negative, and blank controls.

#### 2.8.2 Strip test for Bar protein

To confirm stable protein expression, PCR-positive plants were further assayed using commercial Bar protein quick test strips (E-Cloud Biotech, Wuhan, China) following the manufacturer’s protocol. The confirmed Bar-expressing plant, wild-type plant, and ddH_2_O were used as the positive, negative, and blank controls, respectively.

### 2.9 Statistical analysis

Data from all tissue culture and genetic transformation experiments were collected from three independent biological replicates. Each replicate consisted of 100–120 uncontaminated explants, calli, or shoots. The following parameters were calculated:

Induction Frequency (%) = Number of explants producing embryogenic callus/Total number of explants × 100. This parameter was used to estimate the capacity of explants to form embryogenic callus.

Regeneration Frequency (%) = Number of calli producing shoots/Total number of calli × 100. This parameter was used to measure the proportion of calli that initiated regeneration.

Regeneration Index = Total number of regenerated shoots/Number of differentiated calli × 100. This parameter was used to measure the shoot proliferation efficiency from regenerating calli.

Rooting Frequency (%) = Number of shoots developing roots/Total number of shoots × 100.

Relative Growth (%) = (Final fresh mass–Initial fresh mass)/Initial fresh mass × 100. This parameter was used to estimate the growth response of callus to PPT selection.

Transformation Efficiency (%) = Number of transgenic events/Total number of calli infected × 100. PCR-positive plantlets derived from the same original callus were considered a single transgenic event.

All data are represented as the means ± standard deviation (SD) with three independent replicates. Differences between groups were determined by one-way analysis of variance (ANOVA) followed by Duncan’s multiple comparison, or Student’s *t*-test for two-group comparison. Correlation analysis was performed with a two-tailed test. For all the analyses, *p* < 0.05 was considered statistically significant.

## 3 Results

### 3.1 An efficient tissue culture protocol for Black Cheribon

**Figure 2:**
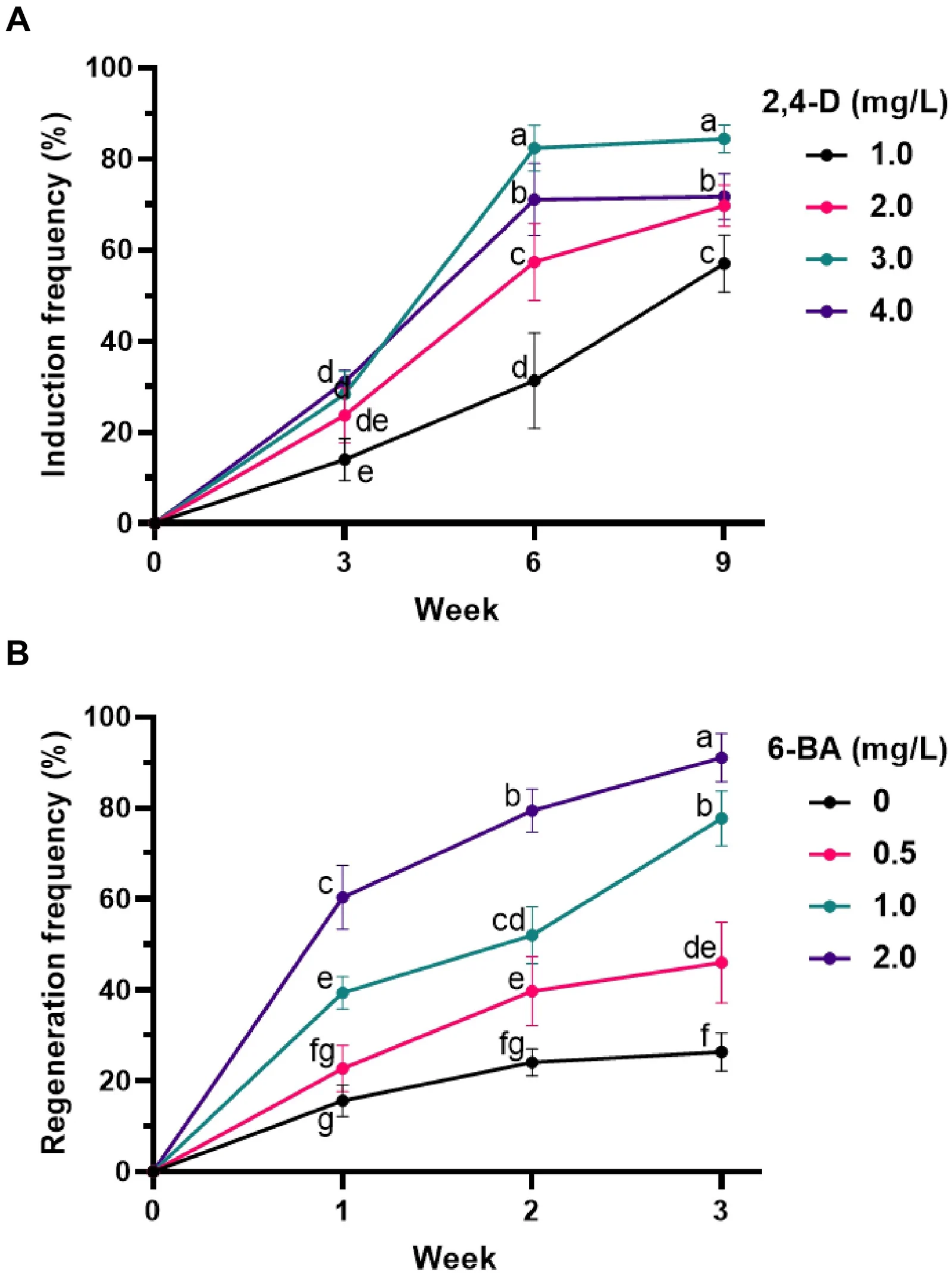
**E**ffect of different concentrations of 2,4-D and 6-BA on embryonic callus induction and regeneration of Black Cheribon. **(A)** Time course of embryonic callus induction frequency under varying 2,4-D concentrations. Leaf discs were used as explants, and data were recorded every three weeks. **(B)** Weekly dynamics of embryonic callus regeneration frequencies at different 6-BA concentrations. Data are shown as means ± SD (n = 3). Data were analyzed by Duncan’s method. Different lowercase letters indicate significant differences at *P* < 0.05.

To establish an efficient regeneration protocol for Black Cheribon, we first evaluated the effect of 2,4-D concentration on embryogenic callus induction. Over a nine-week induction period, the induction frequency increased with both culture duration and 2,4-D concentration (**Figure 2A**). The highest induction frequency for each 2,4-D concentration was observed at the nine-week time point. The total maximum induction frequency of 84.33% was achieved with 3.0 mg/L 2,4-D after nine weeks. However, a comparable frequency of 82.33% was recorded after only six weeks at the same concentration, with no statistically noteworthy difference between the six- and nine-week results. Therefore, culture on medium supplemented with 3.0 mg/L 2,4-D for six weeks was identified as the most efficient protocol for embryogenic callus production in Black Cheribon.

**Figure 3:**
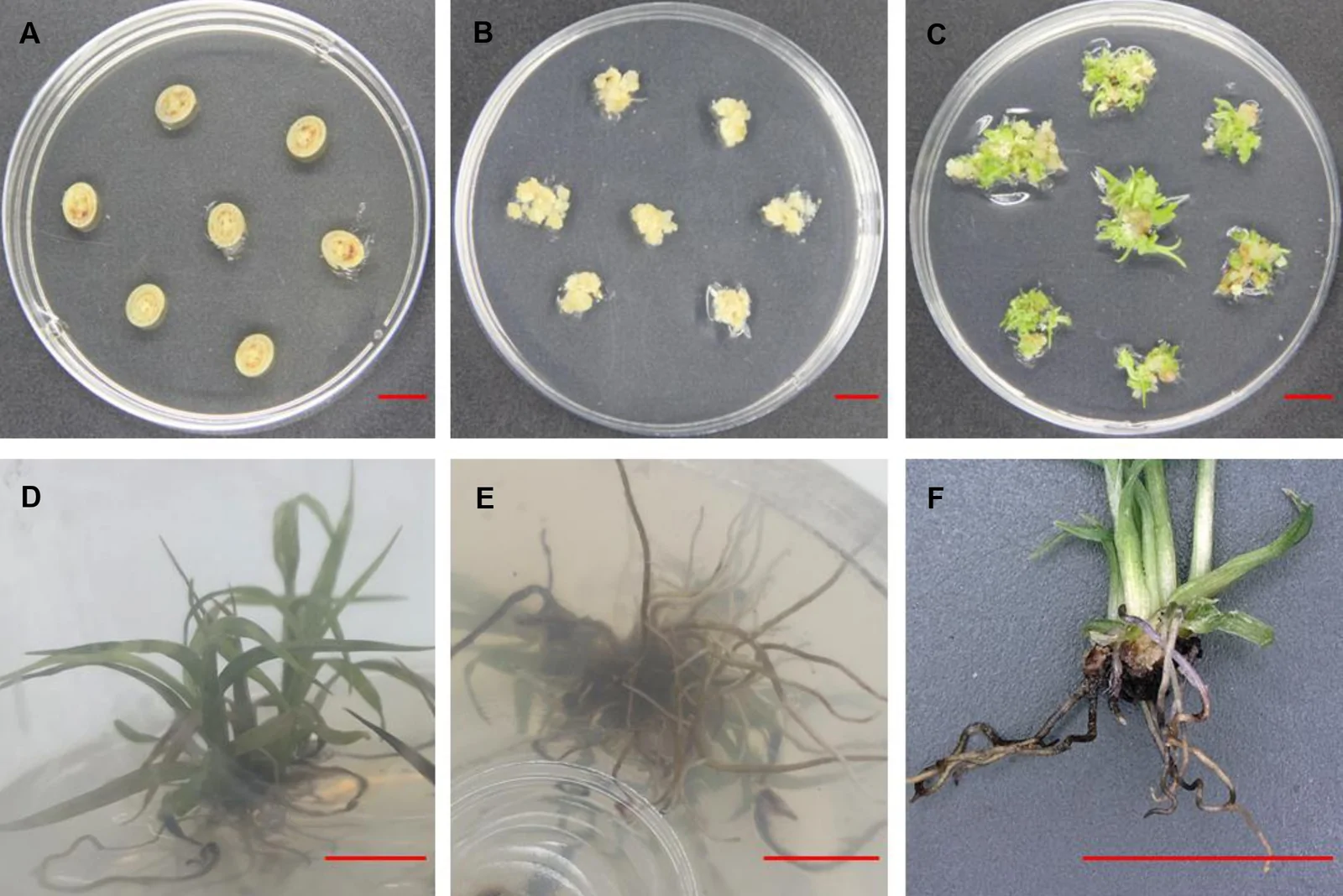
Tissue culture and regeneration of Black Cheribon. **(A)** Leaf-disc explants of Black Cheribon; **(B)** Embryogenic calli. **(C)** Differentiated embryogenic calli. **(D)** Regenerated shoots. **(E)** Rooting of regenerated shoots **(F)** Root system of regenerated plantlet. Scale bar = 1 cm.

The regeneration capacity of the embryogenic calli was then assessed by growing them on media containing different 6-BA concentrations for three weeks (**Figure 2B**). Embryogenic calli showed high regeneration potential. A basal regeneration frequency of 26.33% was found even in hormone-free medium, showing high intrinsic embryogenic potential in this genotype. The frequency of shoot regeneration was also significantly improved on 6-BA supplementation, wherein the highest frequency over 91% was obtained with medium supplemented with 2.0 mg/L 6-BA. From these optimized conditions, a full tissue culture system was developed, and the regeneration of shoots was achieved in 12 weeks (**Figure 3**). In this protocol, leaf disc explants were cultured on CIM (**Table 1**), yielding abundant high-quality embryogenic calli after a six-week incubation in darkness (**Figures 3A, B**). Later, a three-week culture on RM (**Table 1**) under a light cycle promotes efficient shoot differentiation (**Figure 3C**). Transferring the shoots to RM for three additional weeks resulted in the development of plantlets with well-developed root systems (**Figures 3D–F**).

### 3.2 Stage-dependent PPT sensitivity of Black Cheribon

**Figure 4:**
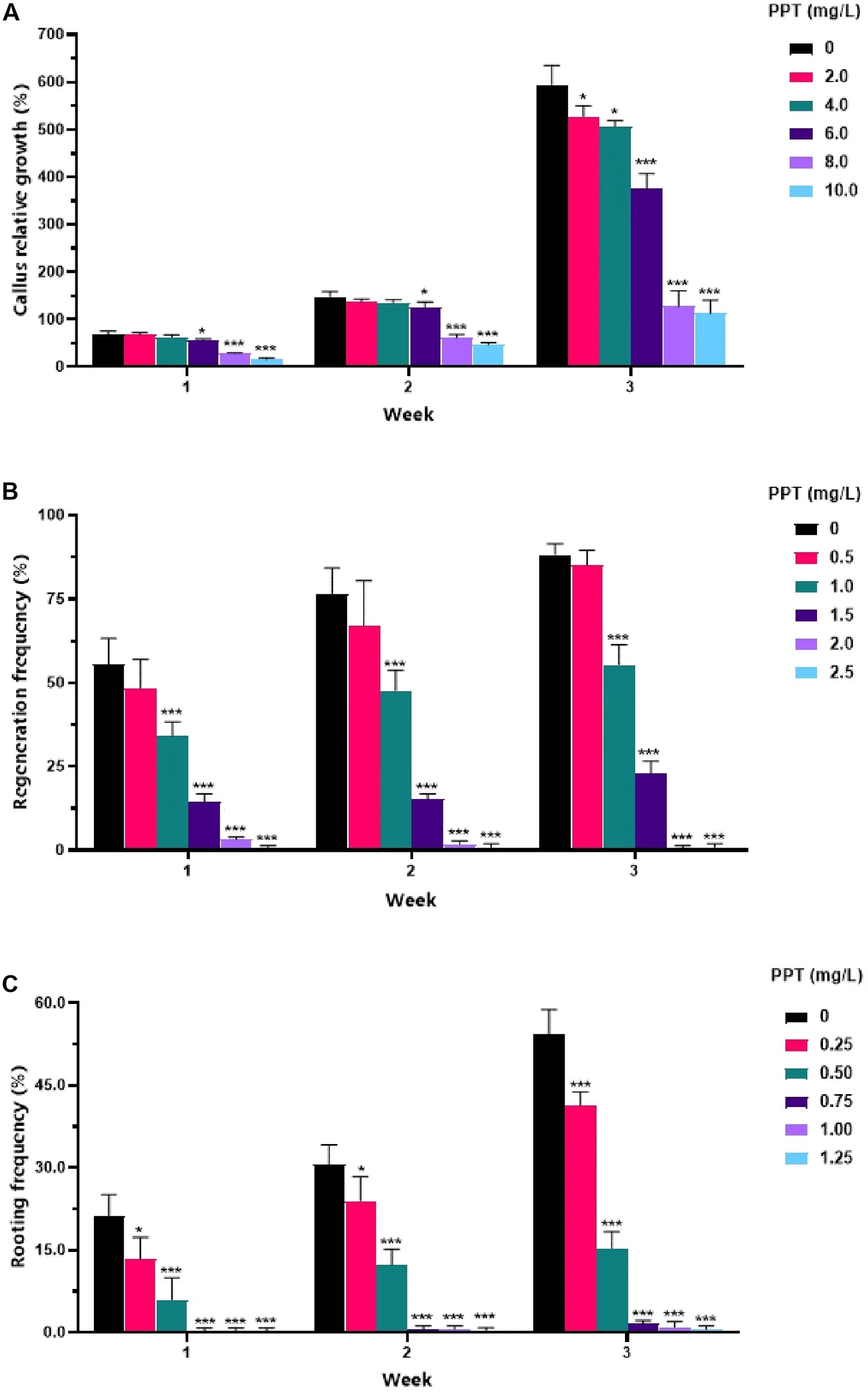
PPT dose response of Black Cheribon at different stages. **(A–C)** Show the dose response of Black Cheribon to PPT at the stages of callus proliferation, callus differentiation, and root induction, respectively. Data are shown as means ± SD (n = 3). We determined the weekly variations between the control (0 mg/L) and the PPT treatments using Dunnett’s test. *P < 0.05; ***P < 0.001.

During the callus proliferation stage, Black Cheribon exhibited significant tolerance to PPT. Although increasing PPT concentrations progressively inhibited callus growth, complete suppression was not achieved even at the highest concentration tested (10.0 mg/L; **Figure 4A**). Concentrations of 2.0 and 4.0 mg/L failed to induce significant growth inhibition until the third week of culture. By contrast, the PPT concentrations of 6.0 mg/L and higher induced statistically significant inhibition throughout the entire incubation period. So, 6.0 mg/L was identified as the MIC for the callus proliferation stage.

In contrast, the shoot regeneration stage was more sensitive to PPT than the callus proliferation stage. The frequency of callus differentiation declined significantly with increased concentration of PPT (**Figure 4B**). Cells exposed to concentrations of PPT > 1.0 mg/L exhibited significant impairment of differentiation, with virtually complete inhibition being observed at concentrations ≥ 2.0 mg/L. This led to the selection of 2.0 mg/L as the MIC for shoot regeneration. The most PPT-sensitive stage was root development phase (**Figure 4C**). Rooting was also significantly suppressed by even the lowest concentration tested (0.25mg/L). The roots were totally inhibited when the level of PPT exceeded 0.75 mg/L. Thus, 0.75 mg/L was found to be the MIC for rooting.

The MICs for the callus proliferation, shoot differentiation, and root development were determined as 6.0, 2.0, and 0.75 mg/L, respectively, using the dynamic data. This clear gradient indicates a negative correlation between tissue organization level and PPT tolerance for Black Cheribon.

### 3.3 Optimization of *Agrobacterium* infection

**Figure 5:**
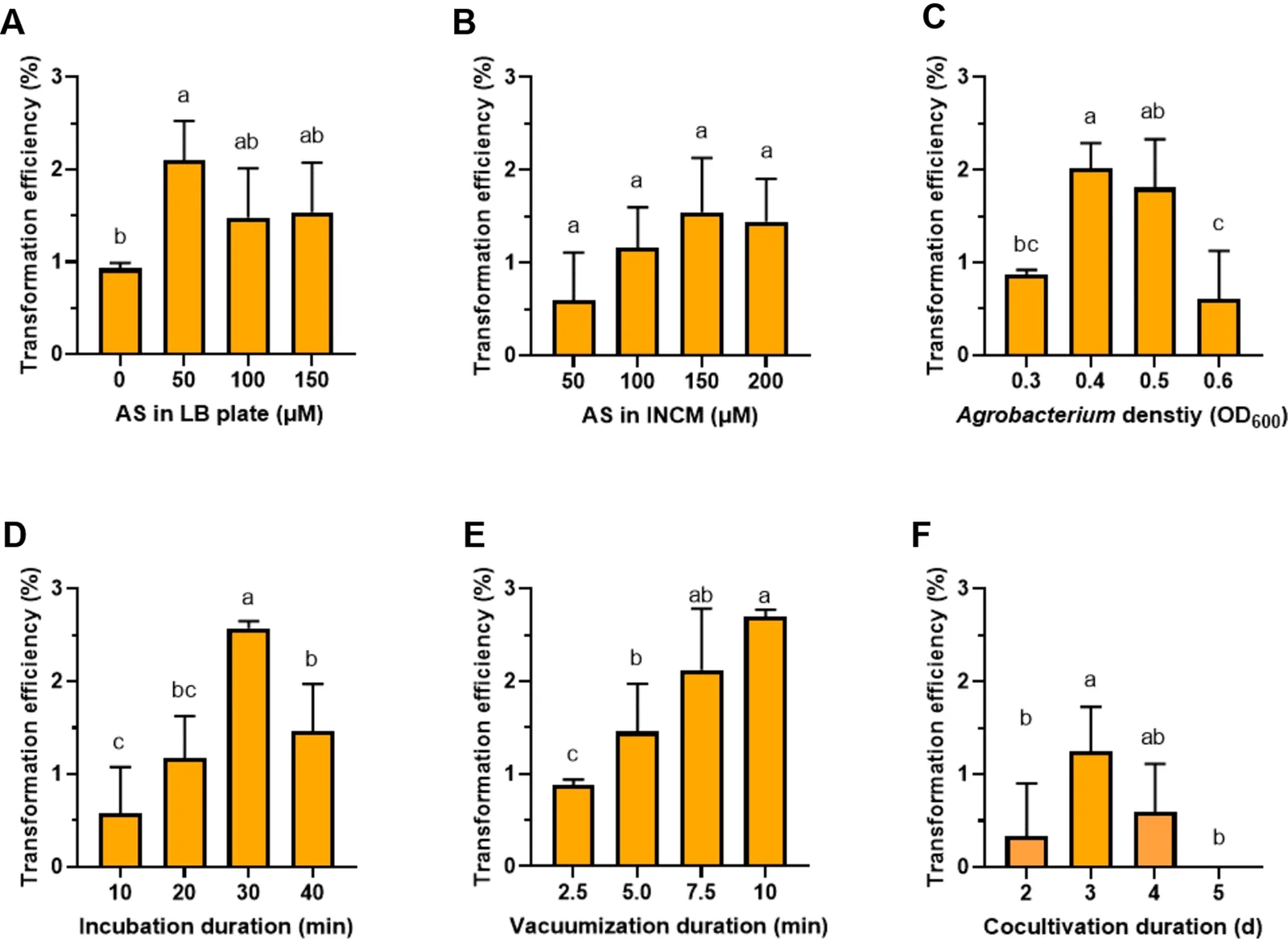
Key factors affecting transformation efficiency in Black Cheribon. **(A–F)** Show the effects of six key parameters on transformation efficiency, including AS in LB plate, AS in INCM, *Agrobacterium* density, infection duration, vacuumization duration, and co-culture duration. Differences in transformation efficiency among the four levels were compared for each factor. Data are shown as means ± SD (n = 3). Different lowercase letters indicate significant differences at *P* < 0.05 (Duncan’s method).

Optimization of the agro-infection procedure for Black Cheribon was undertaken by the one-factor-at-a-time approach. The inclusion of AS in the LB plate used for *Agrobacterium* culture significantly improved transformation efficiency, with the highest value achieved at 50 μM AS (**Figure 5A**). Although the AS concentrations in the INCM liquid did not show statistically significant variation, the highest transformation efficiency was observed at 150 μM (**Figure 5B**). Transformation efficiency was also influenced by *Agrobacterium* density and infection duration, peaking at an OD_600_ of 0.4 (**Figure 5C**) and an infection time of 30 minutes (**Figure 5D**). The treatment time for the vacuumization was also a key variable, as a 10-minute treatment showed the best transformation efficiency (**Figure 5E**). The best co-cultivation period was determined to be 3 days. Growing for 5 days led to large overgrowths of *Agrobacterium* with subsequent callus necrosis and complete plant regeneration failure (**Figure 5F**).

Based on these results, an optimized agro-infection protocol was established by integrating the optimal value for each parameter. This protocol involves culturing *Agrobacterium* on LB plates with 50 μM AS, followed by preparing the bacterial suspension in INCM liquid with 150 μM AS to a density of OD_600_ = 0.4. The calli were immersed in the suspension for 30 min, which includes a vacuum treatment at −35 Kpa for 10 min. The infected calli were then co-cultivated for 3 d at 22 °C.

**Figure 6:**
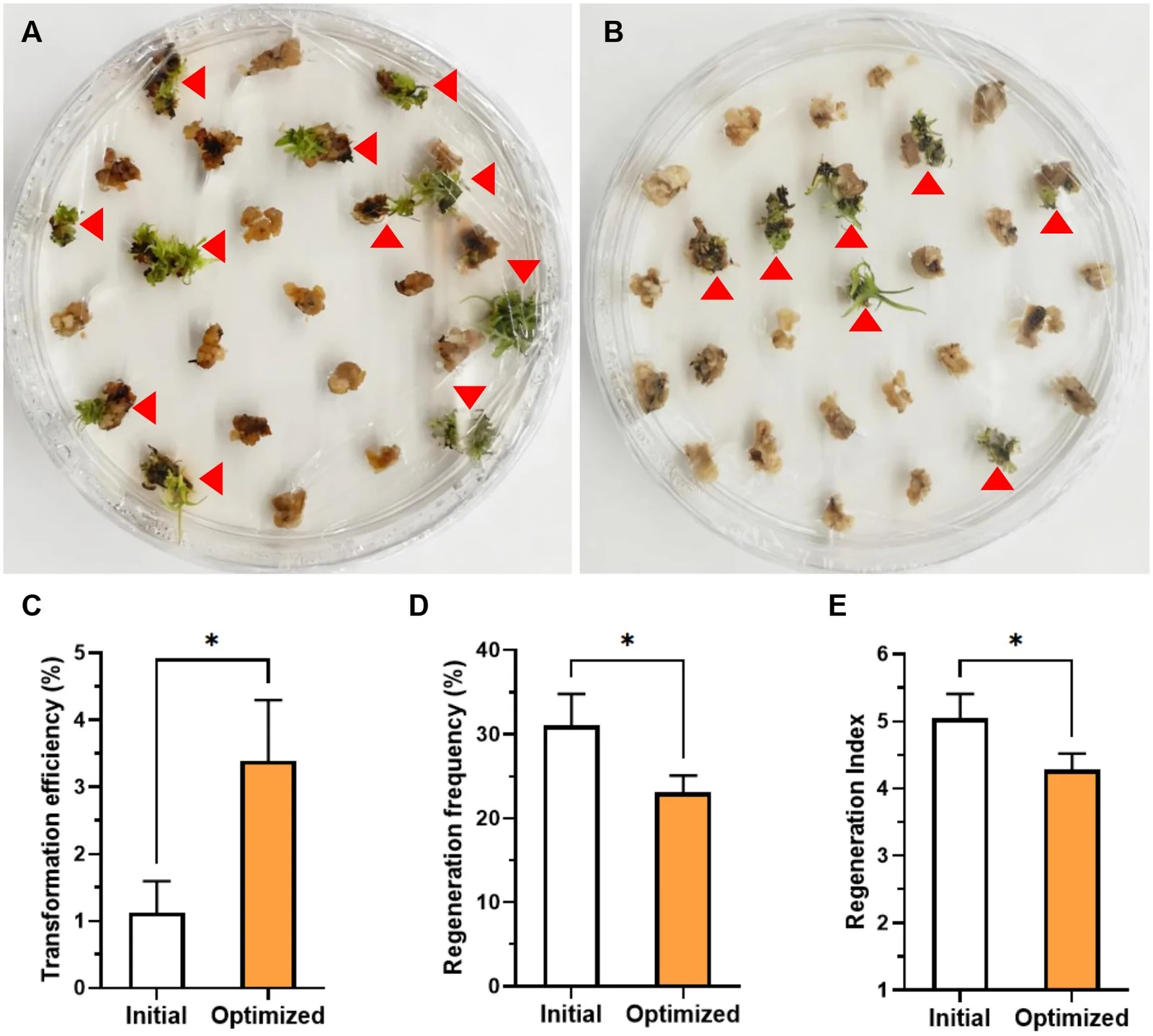
Effect of optimized agro-infection on transformation efficiency and regeneration performance of Black Cheribon. **(A, B)** Show the regeneration of agro-infiltrated calli using initial and optimized methods, respectively. **(C)** Shows the differences between the initial method and the optimized method in transformation efficiency. **(D, E)** Representing differences in regeneration frequency and regeneration index, respectively. The red triangles indicate differentiated calli. Data are shown as means ± SD (n = 3). *P < 0.05 (Student’s *t*-test).

The efficacy of this optimized protocol was then evaluated. Compared to the initial protocol, the optimized method resulted in a significant reduction in regeneration parameters (**Figures 6A, B**). The optimized method increased transformation efficiency nearly threefold, from 1.12% to 3.40% (**Figure 6C**). Additionally, the optimized protocol also negatively affected regeneration frequency and regeneration index (**Figures 6D, E**). Altogether, while the optimized agro-infection procedure enhanced transformation efficiency, it also negatively affected regeneration capacity.

### 3.4 Transformation efficiency varied on different regeneration media

Based on the preceding findings, we hypothesized that the unintended decline in shoot regeneration due to enhanced agro-infection could be mitigated by optimizing the regeneration media, thereby further improving overall transformation efficiency. To examine this hypothesis, we assessed the efficacy of media supplemented with TDZ, which strongly stimulated sugarcane callus regeneration, and citric acid alleviated oxidative damage from agro-infection.

The composition of the regeneration medium significantly influenced shoot regeneration. Overall, TDZ at lower (0.5 mg/L) or medium (1.0 mg/L) concentrations proved beneficial for regeneration frequency. The extent of this improvement was modulated by the concentration of citric acid. The highest regeneration frequency was achieved with medium mRMTP2 (0.5 mg/L TDZ and 200 mg/L citric acid). Unlike that, the highest TDZ concentration (1.5 mg/L) failed to enhance regeneration frequencies at any citric acid level. Regarding the regeneration index, all media except mRMTP1 and mRMTP3 showed improvement to varying degrees. The highest regeneration index (9.10) was achieved with the treatment mRMTP9 (1.5 mg/L TDZ and 300 mg/L citric acid). The transformation efficiency also varied widely among the media. Compared to the control, six of the nine supplemented media showed enhancements. Of these, mRMTP2 had the greatest transformation efficiency of 4.73%.

**Figure 7:**
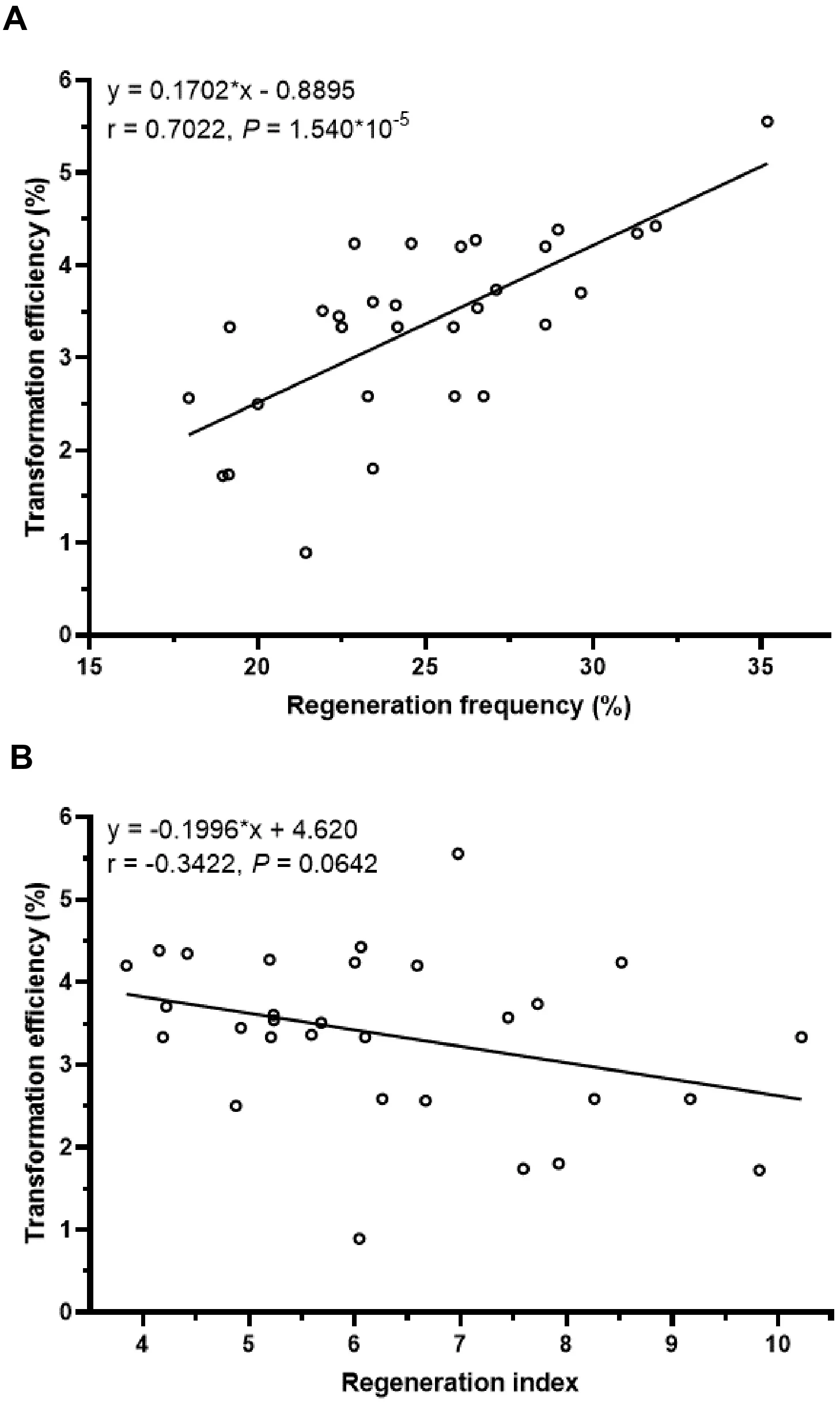
Correlation between transformation efficiency and regeneration performance. **(A)** Relationship between transformation efficiency and regeneration frequency. **(B)** Relationship between transformation efficiency and regeneration index.

According to these results, our findings support our hypothesis that adding TDZ and citric acid to the regeneration medium can improve the genetic transformation efficiency, possibly by inducing shoots. But the data also reveal that enhanced regeneration does not invariably lead to higher transformation efficiency. For instance, mRMTP9 produced the highest regeneration index but not the highest transformation efficiency. To identify which regeneration parameters are more critical, we performed a correlation analysis. This analysis revealed a significant positive correlation between regeneration frequency and transformation efficiency (**Figure 7A**), but a non-significant negative correlation between the regeneration index and transformation efficiency (**Figure 7B**). This finding showed that it would be more critical to increase the regeneration frequencies than to increase the regeneration indices for improvement of the transformation efficiencies.

### 3.5 Effect of selection methods on transformation efficiency

To further improve transformation efficiency through refined selection strategies, we designed three supplementary selection protocols informed by the PPT dose-response profile of Black Cheribon. These three protocols, together with that of the initial method, were comparatively evaluated for their efficacy in generating transgenic plants. We found that the transformation efficiency differed from one protocol to another (**Table 3**). Contrary to expectation, the strict protocol, which rigorously adhered to stage-specific MICs, yielded the lowest transformation efficiency. However, both FlexI and FlexII proved that they could deliver significantly higher efficiencies than the strict approach. Interestingly, no statistically significant differences resulted for the FlexI method compared with the initial protocol, but for the FlexII method, the highest transformation efficiency obtained (7.17%) was the maximum among all experimental conditions. For this reason, FlexII was found to be the most effective selection method for Black Cheribon transformation.

### 3.6 Molecular assay of resistant plants

**Figure 8:**
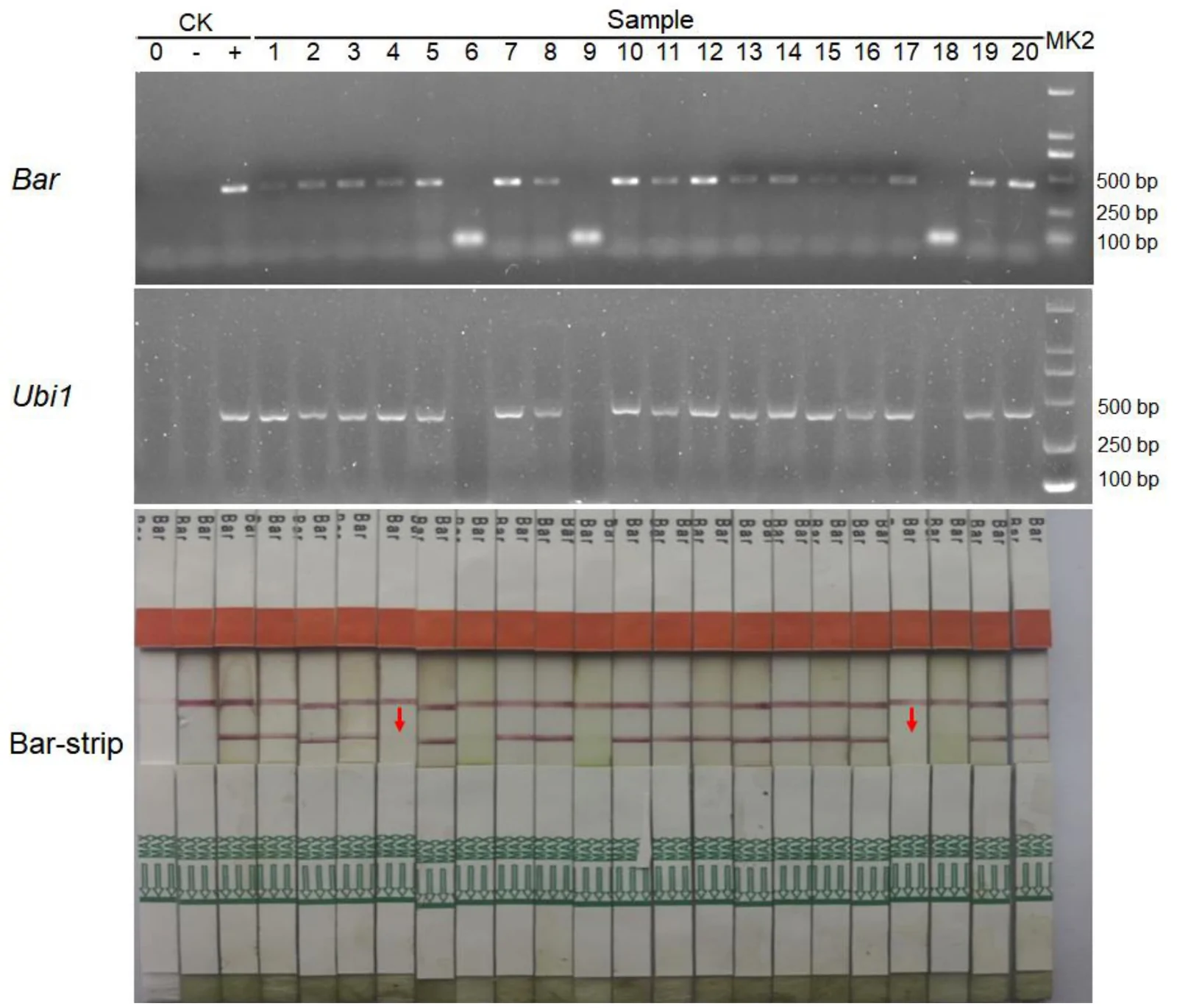
Molecular assay of putative transgenic plantlets of Black Cheribon. Molecular analysis of 20 resistant plantlets is shown. Dual-target PCR was employed for T-DNA detection, yielding amplification of a 489-bp *Bar* gene fragment and a 432-bp *Ubi1* promoter fragment. The expression of the *Bar* gene product was detected using quick test strips for the Bar protein. Red arrows indicate samples that were PCR-positive but strip-negative. CK0 and CK- represent blank controls (ddH_2_O) and negative controls (wild-type Black Cheribon), respectively. CK+, positive control. For PCR detection, CK+ presents the plasmid vector used for transformation. For the bar-strip assay, CK+ presents a previously confirmed strip-positive plant. MK2, Trans2K DNA Marker.

For the validation of this optimized protocol, six independent transformation experiments were performed and 147 putative transgenic plantlets were obtained (**Table 4**). Dual target PCR assay and immunochromatographic Bar protein strips (**Figure 8**) were used to perform the molecular assay. Of these, 127 (86.4%) were found to be transgenic by PCR analysis, with an average transgene integration rate of 85.9% in all experiments. The presence of the intact T-DNA region was verified by amplification of both *Bar* gene (left-border) and *Ubi1* promoter (right-border) regions in these positive plantlets. Bar protein strips detected the transgenic protein in 119 of the 127 PCR-positive plantlets (93.7%), showing successful transgene expression in the majority of the transgenic events at the T_0_ stage.

**Table 4:** Summary of molecular analysis of putative transgenic Black Cheribon plantlets.

Experiment	Putative transgenic plantlets No.					Positivity rate	
	Total	*Bar-*positive	*Ubi1*-positive	Dual-site positive	Bar-strip positive	PCR (%)	Bar-strip (%)
1	18	15	15	15	15	83.33	100
2	23	19	19	19	17	82.61	89.47
3	20	17	17	17	16	85	94.12
4	32	29	29	29	26	90.63	89.66
5	31	27	27	27	25	87.1	92.59
6	23	20	20	20	20	86.96	100
Mean	24.5 ± 5.8	21.2 ± 5.6	21.2 ± 5.6	21.2 ± 5.6	19.8 ± 4.7	85.9 ± 2.9	94.3 ± 4.7
Total	147	127	127	127	119	86.4	93.7

PCR-positivity rate is defined as the proportion of plantlets confirmed by dual-target PCR for *Bar* and *Ubi1*. The Bar-strip positivity rate represents the percentage of PCR-positive plantlets that also tested positive for the Bar protein via immunochromatographic strip assay. Average data are shown as means ± SD of the six independent experiments.

### 3.7 Summary of optimized transformation protocol for Black Cheribon

**Figure 9:**
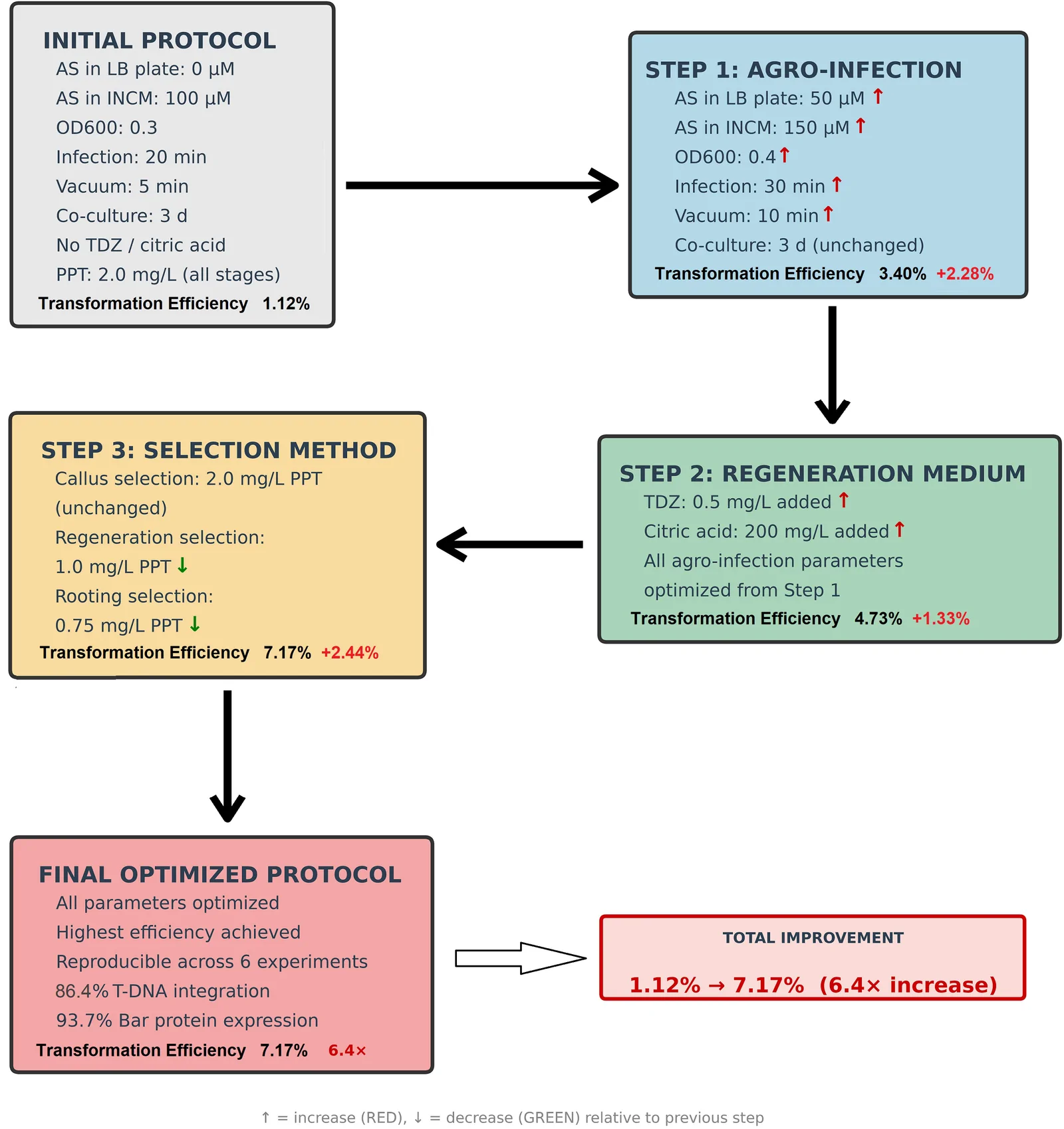
Schematic workflow of the sequential optimization strategy employed to develop the *Agrobacterium*-mediated transformation system for *Saccharum officinarum* cv. Black Cheribon. The workflow represents the three consecutive optimization modules: (1) agro-infection parameters (AS concentrations, bacterial density, infection time, and vacuum duration), (2) supplementation of the regeneration medium with TDZ and citric acid, and (3) selection method (FlexII strategy with stage-specific PPT concentration). Each step of the transformation is presented with its efficiency, with stepwise improvements and cumulative gains highlighted. Arrows indicate the progression from initial protocol through each optimization module to the final optimized protocol. The arrows, (↑) or (↓), are used to represent a change in key parameters at each step, showing whether the parameter increased or decreased compared to the previous step. The overall improvement (from 1.12% to 7.17%) corresponds to a 6.4-fold relative increase in transformation efficiency. The efficiencies displayed are the result of an optimization process that was carried out sequentially, using a one factor at a time approach, and do not reflect the independent contribution of each module.

**Figure 10:**
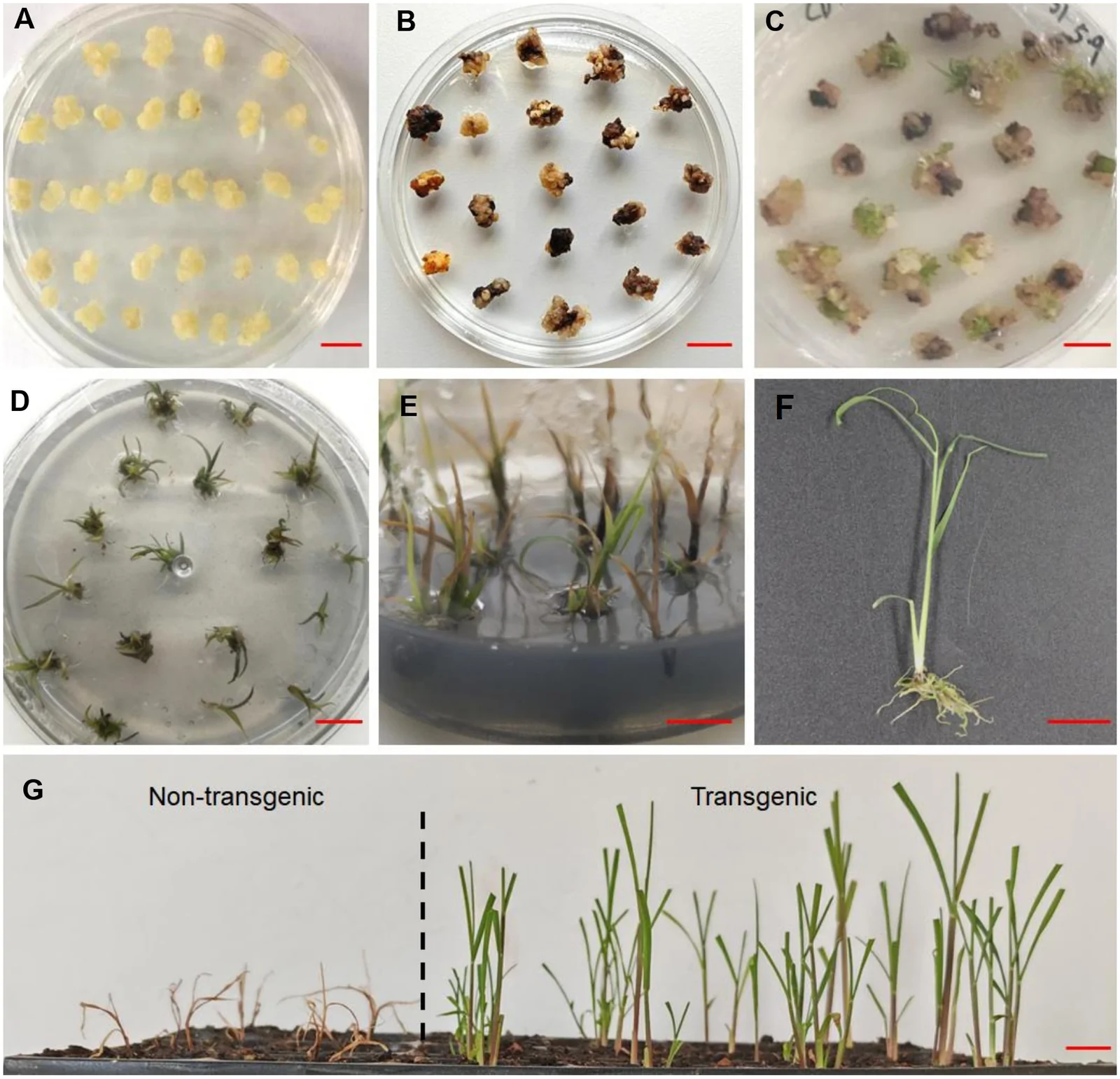
Efficient *Agrobacterium*-mediated genetic transformation for Black Cheribon. **(A)** Embryonic calli of Black Cheribon used for transformation. **(B)** Selection for resistant calli following agro-infection. After PPT selection at 2.0 mg/L for 6 weeks, calli exhibited varying degrees of browning and necrosis. **(C)** Selection at the differentiation stage. Following 6 weeks of selection on 1.0 mg/L PPT, a part of the resistant calli underwent differentiation. **(D, E)** Selection at the rooting stage. Resistant shoots isolated from calli were subjected to selection on 0.75 mg/L PPT. After 6 weeks, some of the non-transgenic shoots showed necrosis. **(F)** A resistant plantlet exhibiting normal growth and development after the sequential PPT selection process. **(G)** Herbicide resistance assay. Plantlets were sprayed with 160 mg/L Basta solution, with non-transgenic tissue-cultured plantlets serving as controls. After 2 weeks, non-transgenic plantlets died, whereas the majority of surviving resistant plantlets were confirmed as transgenic. Scale bar = 1 cm.

By systematically optimizing the agro-infection, regeneration medium, and selection method, we established an efficient AMT protocol for *S. officinarum* using Black Cheribon as the model genotype (**Table 5**; **Figure 9**). In this optimized protocol, embryogenic calli, induced on CIM medium (**Table 1**) for six weeks (**Figure 10A**), were agro-infected using an enhanced method. Following a one-week recovery on CIMT medium (**Table 1**), the calli were subjected to a six-week selection period on CIMTP medium (**Table 1**) supplemented with 2.0 mg/L PPT (**Figure 10B**). Resistant calli were returned to mRMTP2 medium (**Table 2**) containing 1.0 mg/L PPT, where they were maintained for six more weeks to encourage shoot regeneration under selective pressure (**Figure 10C**). The resistant shoots were then grown in RTMTP media (**Table 1**) containing 0.75 mg/L PPT to regenerate plantlets with roots (**Figures 10D–F**). The optimized protocol greatly enhanced the genetic transformation efficiency from 1.12% to 7.17% (**Table 5**). The resulting transgenic lines were significantly more tolerant to the herbicide Basta (**Figure 10G**). PCR analysis confirmed T-DNA integration in 127 (86.4%) of 147 putative transformants, while *Bar* protein expression was detected in 119 (93.7%) of the 127 PCR-positive plants.

**Table 5:** Comparison of the initial and optimized protocols for *Agrobacterium*-mediated genetic transformation of Black Cheribon.

Subject	Parameter	Initial	Optimized
Agro-infection	AS in LB plate (μM)	0	50
	AS in INCM (μM)	100	150
	*Agrobacterium* density (OD_600_)	0.3	0.4
	Incubation duration (min)	20	30
	Vacuumization duration (min)	5	10
	Co-culture duration (d)	3	3
Regeneration medium	TDZ (mg/L)	0	0.5
	Citric acid (mg/L)	0	200
Selection method	PPT in callus selection (mg/L)	2	2
	PPT in regeneration selection (mg/L)	2	1
	PPT in rooting selection (mg/L)	2	0.75
Transformation efficiency (%)		1.12	7.17

## 4 Discussions

### 4.1 Black Cheribon is a model *S. officinarum* genotype with high tissue culture performance

*S. officinarum* is an important species for the breeding of modern sugarcane and has played a decisive role in breeding programs. Black Cheribon is the most commonly used *S. officinarum* parent in cross-breeding among these genotypes (Deren, 1995). This study also revealed its biotechnological applicability as Black Cheribon was found to be highly responsive to tissue culture. A conventional tissue culture protocol was found effective for inducing embryonic callus and achieving high-frequency shoot regeneration in Black Cheribon, requiring no specialized media or treatments. Although literature on its tissue culture performance is limited, the two existing reports consistently identify Black Cheribon as possessing superior tissue culture response compared to other *S. officinarum* genotypes such as Badila (Van der Vyver, 2010) and NG77-69 (Pillay, 2013). The consistent findings from various experiments strongly suggest that high tissue culture response is a genetically intrinsic trait in Black Cheribon.

This is because all current model genotypes for the genetic transformation of sugarcane are modern cultivars, such as ROC22 or CP88-1762. The model genotypes of sugarcane currently used for genetic transformation are all modern cultivars, for example, ROC22 and CP88-1762. Their superior tissue culture ability can be attributed to their long history of being continuously selected for agronomic traits related to their high regeneration capacity, as well as for conferring complex genomes (Li et al., 2003). But, less domesticated wild germplasm, however, often has a less pronounced tissue culture response, but with a comparatively simple genome (Silva et al., 2020; Benlioğlu et al., 2025). This natural balance between the responsiveness of the tissue culture and the complexity of its genome is what makes Black Cheribon so unique. Black Cheribon is an excellent tissue culture-responsive plant with a relatively simple genome representing wild germplasm of *S. officinarum*. These overall characteristics make Black Cheribon a potential model genotype for biotechnology research of *S. officinarum*.

### 4.2 Optimizing agro-infection parameters to enhance transformation

Agro-infection affects the plant’s transformation efficiency by triggering the genetic transformation process. Based on the developed mechanistic approach of AMT (De Saeger et al., 2021) this study systematically optimized the agro-infection protocol for Black Cheribon by focusing on three crucial steps: vir gene activation, bacterial adhesion, and T-DNA integration. Accordingly, our strategy was aimed at each of these steps directly. The vir genes were activated by optimization of AS levels in the *Agrobacterium* culture media (LB plates and INCM). The *Agrobacterium* density, incubation time and duration of vacuum infiltration were optimized to promote bacterial adhesion to the plant cell. Finally, we promoted T-DNA integration by optimizing the co-cultivation period. This approach resulted in a more than threefold increase in the transformation efficiency of Black Cheribon.

An interesting finding from our optimization was the absence of significant differences in transformation efficiency across the tested AS concentrations (50-200 μM) in the INCM. This was unexpected since sugarcane, a monocot and non-natural host for *Agrobacterium*, requires supplemental AS for *vir* gene expression, which is known to be concentration-dependent (Stachel et al., 1985; Shimoda et al., 1990; Sutradhar and Mandal, 2023). We suggest that this is because the INCM has a high glucose content (20 g/L). The high glucose concentration was likely to have buffered the effect of the varied AS levels as far as transformation efficiency was concerned, since it has been found that reducing monosaccharides potentiates vir gene expression (Shimoda et al., 1990, 1993; Sutradhar and Mandal, 2023). This suggests a synergistic effect between AS and reducing sugars that may be applied to improve agro-infection techniques for sugarcane transformation. The transformation efficiency was increased by optimizing our protocol through adjustment of infection parameters such as bacterial density, vacuum infiltration, infection duration and longer co-culture period. But it is well known that maximum infection intensity does not lead to improved transformation. In general, over-stressing agro-infection will have a negative impact on the viability of host tissue, resulting in a reduced transformation efficiency (Pitzschke, 2013; Azizi-Dargahlou and Pouresmaeil, 2024) In line with this principle, we witnessed this trade-off directly in our study. Moderate, not maximal, levels of most parameters were found to be optimal to maximize the efficiency of each parameter. Furthermore, increasing the co-cultivation time beyond a certain limit was detrimental to the regeneration of transgenic plants. Similar results were observed for other plant species like *Glycine max* (Li et al., 2017), *Brassica napus* (Dai et al., 2020), *Cinnamomum camphora* (Zhang et al., 2023), and *Brassica juncea* (Fu et al., 2024). These results suggest that a systematic balance between infection intensity and callus viability to create a reliable protocol is needed.

### 4.3 Overcoming the trade-off between agro-infection and regeneration

The optimized agro-infection protocol enabled efficient transformation, while simultaneously reducing the callus regeneration capacity. It is interesting to note that this trade-off also exists in other monocots (Zhang et al., 2013; Nguyen et al., 2020; Bhatt et al., 2021). The impairment of regeneration may be due to the cumulative effects of biological stress resulting from the presence of the pathogen (*Agrobacterium*) and physical stress from the infiltration process, such as a vacuum infiltration system. This stress simultaneously activates ROS burst and programmed cell death. This has a negative impact on the viability and differentiation capacity of the callus (Patel et al., 2013; Zhang et al., 2013; Pang et al., 2025).

To counteract this, TDZ and citric acid were incorporated into the regeneration medium. TDZ was chosen because it has been proven to act as a cytokinin, which can be 10–100 times more effective than 6-BA or KT, and possesses an auxin-like activity, so stimulating cell division and auxin-controlled pathways for organogenesis (Lakshmanan et al., 2005; Brugière et al., 2008). Previous studies reported that TDZ possesses significantly higher morphogenic activity than conventional cytokinins, thereby promoting cell division, meristem initiation, and shoot organogenesis (Huetteman and Preece, 1993; Murthy et al., 1998; Wang et al., 2025). To combat oxidative damage and prevent phenolic browning, citric acid was used as an antioxidant, which has a dual function as an ROS scavenger and metal ion chelator, thereby reducing oxidative stress, limiting phenolic oxidation, and preserving tissue viability during regeneration (Tahjib-Ul-Arif et al., 2021). It is remarkable that the regeneration capacity was completely restored in the modified medium, with 0.5 mg/L TDZ and 200 mg/L citric acid, in comparison with the initial medium. The observed improvement implies that augmentation of organogenic competence, along with mitigation of oxidative damage, was a significant component of the recovery of regeneration potential after agro-infection. The modified medium also further enhanced the transformation efficiency. These findings support the concept that optimizing the balance of plant growth regulators and antioxidant protection can be an effective strategy for overcoming regeneration bottlenecks associated with AMT. The morphogenetic factors such as WUS (Xiao et al., 2018; Che et al., 2022), BBM (Deng et al., 2009; Wang et al., 2023), or GRF-GIF chimeras (Debernardi et al., 2020; Bull et al., 2023) are expressed ectopically, but perform similar functions with a divergent mechanism meant to enhance cellular totipotency and differentiation. Genetic approaches like these, combined with the optimized medium developed in this study are promising for synergic approaches in the development of a more stable and efficient transformation system in sugarcane. Shoot regeneration has been recognized before as another composite trait involved in genetic transformation (Smith and Hood, 1995; Maren et al., 2022; Azizi-Dargahlou and Pouresmaeil, 2024), but it requires further dissection. Here, the capacity of regeneration was subdivided into two parameters: regeneration frequency (percentage of calli that regenerate) and regeneration index (number of shoots per regenerating callus). The regeneration frequency was determined to influence the transformation efficiency more than the regeneration index. This is because each independent regeneration event provides an individual opportunity for stable transformation. While shoots that regenerate from a single callus are more likely to be clonal (Capriotti et al., 2023; Carmona-Martín et al., 2025) and do not provide any further independent chance for transformation events to occur. So, the initial goal in genetic transformation should be to achieve as many independent callus events as possible rather than obtaining the maximum number of responsive calli to regenerate shoots. The conclusion is based on the traditional optimization framework, but it is expected to be more widely applicable to more advanced systems. Such as those based on morphogenetic factors, because the promotion of regeneration is a universally applicable strategy in genetic transformation optimization.

### 4.4 The trade-off between selection pressure and cell viability

The development of an accurate selection system is important for optimizing transformation efficiency since it eliminates non-transformants (Joyce et al., 2009) while not overly suppressing callus formation and differentiation. The dose-response analysis performed in this study aimed to find an optimal selection system for *Bar*-PPT for Black Cheribon. This approach is consistent with established practices in other crops, such as the *ahas*-imidazolinone system in cotton (*Gossypium* spp.) (Aragão et al., 2005), the *PMI*-mannose system in maize and wheat (*Triticum aestivum*) (Wright et al., 2001), and the *NptII*-neomycin system in sugarcane (Joyce et al., 2009). Although these previous studies, along with the current report, indicate that dose-response analysis is widely applicable for optimization of selection systems, our approach differs in its selection of assessment metrics. Callus fresh weight is another important measurement in these studies, as it is a general measure of the biomass that is gained during callus proliferation. So, to obtain a more comprehensive view across the entire development process, we employed stage-specific indicators. While fresh weight was used to assess callus proliferation, we precisely monitored regeneration frequency during shoot differentiation and rooting rate during root development. Therefore, our stage-specific metrics are likely more direct measures of successful organogenesis from putatively transformed tissue.

In the present study, applying a sub-inhibitory selection pressure to enhance transformation efficiency was effective, while a previously described MIC was ineffective under extreme selection pressure. This finding contrasts with reports of other studies in species like (*Solanum lycopersicum*) (Ahmed et al., 2023) and liverwort (*Marchantia polymorpha*) (Poormassalehgoo et al., 2025), that recommends selection of plants based on MIC to maximize transformation efficiency, such a strategy is suggested for sugarcane (Wang et al., 2017). However, the superiority of the strict MIC selection, in these reports, is not always unequivocal as there is no comparative assessment with sub-MIC protocols. The comparative analysis of various selection approaches clearly showed the advantage of FlexII in our study, which was based on a sub-MIC strategy. Recently, Wang et al., 2024 developed an efficient CP4-EPSPS-glyphosate transformation system in sugarcane cultivar ROC22. The highest transformation efficiency was achieved using their protocol of 50% MIC for the resistant callus and 25% MIC for the following regeneration. Together, these findings suggest that sub-inhibitory selection pressure may represent a promising strategy for improving transformation efficiency in sugarcane.

The inability of the strict MIC-based selection is hypothesized to be a direct result of the physiological injury undergone at the time of agro-infections in the calli. The transcriptomic studies revealed a swift increase in ROS accumulation and a significant decrease in stress tolerance in the infected tissues (Willig et al., 2018; Li et al., 2021). In plants, this conserved stress response (Willig et al., 2018) indicates that such physiological changes are likely to occur in Black Cheribon callus following the infection. Similar physiological alterations probably occur in Black Cheribon callus after infection. These alterations would likely reduce the tolerance of callus to PPT, thereby rendering an MIC established using healthy, uninfected callus excessively cytotoxic under post-transformation conditions. Thus, we propose a promising approach for further optimizing the selection system, the determination of pressure directly in the agro-infected callus compared to the uninfected callus. This method takes into consideration the physiologically stressed state of the callus during transformation and would help in making more reliable and effective selection.

In summary, we established a highly efficient *Agrobacterium*-mediated transformation system for *S. officinarum* using Black Cheribon as the model genotype. Despite these advances, several aspects warrant further investigation. First, the optimization of agro-infection, regeneration, and selection was performed sequentially, and for the agro-infection, a one-factor-at-a-time experimental design was employed. Although this approach yielded an efficient protocol, it does not allow quantification of the relative contributions of individual parameters or detection of potential interactions, thus limiting the likelihood of identifying a global optimum. Future work employing multifactorial strategies, such as factorial design, response surface methodology (RSM), or machine-learning-assisted optimization, may provide deeper insights into factor interactions and help define globally optimal conditions for transformation efficiency (Missaghi et al., 2024). Second, molecular characterization in this study was confined to confirming transgene integration and expression in T_0_ generation. In vegetatively propagated crops such as sugarcane, transgene integration is generally mitotically stable. However, transgene expression may vary across clonal generations due to epigenetic silencing. Therefore, long-term, multi-generation trials conducted across multiple environments are needed to verify the stability of transgene expression. Finally, the current protocol was optimized specifically for the Black Cheribon genotype with *Bar*-PPT selection system. Given the well-known genotype dependence of regeneration and transformation efficiency in sugarcane, the generalizability of this protocol to other *S. officinarum* genotypes with different selection markers needs to be systematically evaluated.

## 5 Conclusions

This study has led to the development of a highly efficient *Agrobacterium*-mediated genetic transformation system for Black Cheribon (*Saccharum officinarum)*. Beyond the use as a foundational hybrid parent for sugarcane breeding, the high tissue culture competence of this genotype has been confirmed in our work, making it a valuable, highly responsive model genotype for sugarcane biotechnology. The genetic transformation efficiency was step-wise improved by optimizing the *Agrobacterium* infection protocol, the composition of the regeneration medium and the selection method, from an initial efficiency of 1.12% to a final efficiency of 7.17%. The transgenic plants produced by this optimized protocol had stable transgene expression and intact T-DNA inserts. The results emphasize the significance of high regeneration frequency and show that a flexible selection pressure (FlexII) is more effective in improving the sugarcane transformation. The developed protocol provides a basic platform for advanced functional genomics and bioengineering approaches, particularly using CRISPR/Cas-based gene editing, to accelerate sugarcane breeding. The identified optimization principles in this study may serve as a useful reference for the development of transformation systems in other sugarcane germplasm, but further testing with different sugarcane genotypes and selectable marker systems will be necessary to determine their broader applicability.

## Data Availability

The original contributions presented in the study are included in the article/supplementary material. Further inquiries can be directed to the corresponding authors.
